# Closed cervix is associated with more severe illness in dogs with pyometra

**DOI:** 10.1186/s12917-016-0924-0

**Published:** 2017-01-05

**Authors:** Supranee Jitpean, Aime Ambrosen, Ulf Emanuelson, Ragnvi Hagman

**Affiliations:** 1 Department of Surgery and Theriogenology, Faculty of Veterinary Medicine, Khon Kaen University, Khon Kaen, 40002 Thailand; 2 Department of Clinical Sciences, Swedish University of Agricultural Sciences, Box 7054, SE-750 07 Uppsala, Sweden

**Keywords:** SIRS, Sepsis, Hematology, Uterine infection, Dogs

## Abstract

**Background:**

Pyometra, a life-threatening bacterial infection of the uterus, is classified as open or closed depending on the functional patency of the cervix i.e. presence or absence of vaginal discharge. In closed cervix pyometra, pus and bacterial products accumulate in the uterus, which is thought to induce a more severe illness. The aim of this study was to investigate whether disease severity or outcome differed in dogs with open or closed cervix pyometra.

**Results:**

Prospectively collected data from 111 female dogs diagnosed with pyometra at the University Animal Hospital, Swedish University of Agricultural Sciences, Uppsala, intermittently during 2005–2012 was analyzed. Seventy-two dogs (65%) had open cervix, whereas 39 dogs (35%) had closed cervix. Differences between the two groups were explored by Wilcoxon Two Sample Test for continuous variables and Chi-square or Fisher’s exact test for categorical variables. *P* < 0.05 was considered significant.

In dogs with open cervix the median age was 9.0 years and the median weight 26.0 kg. In dogs with closed cervix the median age was 9.6 years and the median weight 25.0 kg, with no significant differences between the groups (*p* = 0.69 and 0.24, respectively). Five dogs (4.5%) died, all with open cervix, and 16 dogs (14%) had complications.

The general physical condition was moderately or severely depressed in 30% (21/71) of dogs with open cervix (severely depressed in 4 dogs, moderately depressed in 17 dogs) and in 56% (22/39) of dogs with closed cervix (severely depressed in 3 dogs, moderately depressed in 19 dogs). The general physical condition was mildly depressed in 41 dogs with open cervix and 16 dogs with closed cervix, whereas it was normal in nine dogs with open cervix and one dog with closed cervix. None of the included dogs had very severely depressed general physical condition or were non-responsive.

Leukocytosis, neutrophilia, monocytosis and moderately to severely depressed general condition was more commonly found in dogs with closed cervix (*p* = 0.003, *p* = 0.008, *p* = 0.003 and *p* = 0.006, respectively).

Sepsis was more commonly present in closed cervix pyometra (77%, 30/39 dogs) compared to open cervix pyometra (51%, 36/71 dogs) (*p* = 0.007). Presence of prolonged postoperative hospitalization did not differ significantly between the two groups.

**Conclusions:**

In dogs with closed cervix, sepsis was more common, the general physical condition more often moderately to severely depressed and leukocytosis, neutrophilia and monocytosis more frequently found. The results showed that closed cervix was associated with a more severe illness than open cervix at admission but not with poorer outcome as measured by postoperative hospitalization. These findings may be clinically valuable for optimizing monitoring and treatments in dogs with the disease.

## Background

One of the most frequent reproductive organ disorders in female dogs is pyometra, which affects on average nearly 20% of all intact bitches before 10 years of age [1]. The disease is associated with inflammation and infection of the uterus leading to generalized illness. The disease often generates systemic inflammatory response syndrome (SIRS), defined as sepsis when initiated by infection [2–5]. Gram-negative bacteria are most commonly isolated from pyometra uteri and foremost *Escherichia coli* (*E. coli*). Moreover, Gram-negative bacteria contain endotoxin which is released into the circulation during bacterial growth and death, and endotoxin is a potent inducer of inflammation [6, 7]. Effects of sepsis and endotoxaemia can further cause multi-organ dysfunctions in pyometra, but despite being a potentially life-threatening illness, the mortality is relatively low, 3–10% [8, 9]. The safest and most efficient treatment is surgical removal of the infected uterus and ovaries, i.e. ovariohysterectomy (OHE). Pyometra is classified clinically as closed (without vaginal discharge) or open cervix pyometra (with vaginal discharge) depending on the functional patency of the cervix. If the cervix is closed, the pus and bacterial products remain in the uterus, which is believed to lead to a more serious illness compared to when there is some drainage via the cervix [10]. The preliminary diagnosis of pyometra is determined by case history data, physical examination findings and laboratory test results in combination with radiography or/and ultrasonography showing a fluid-filled enlarged uterus. Clinical signs commonly present in pyometra include depression, anorexia, polydipsia/polyuria, vomiting and vaginal discharge [9, 11, 12]. Leukocytosis, neutrophilia with a left shift, monocytosis and anaemia are frequently observed [4, 9, 13]. Elevated creatinine and blood urea nitrogen [11], proteinuria, hypoalbuminemia, hypercholesterolaemia, increased serum alkaline phosphatase (ALP) [14, 15], and coagulation impairment [16] have also been reported. In cases presented without vaginal discharge, it may be more difficult to recognize the disease at an early stage because most other signs of illness are unspecific. Consequently the disease could have been progressing for a longer time before treatment when being diagnosed in dogs with closed cervix pyometra which may contribute to the general assumption of a more severe illness in such cases [17]. The aim of this study was to test the hypothesis that closed cervix leads to more severe illness by investigating whether disease severity and outcome or presence of complications differ between dogs with open or closed cervix pyometra as determined by clinical and laboratory findings.

## Results

### Open versus closed cervix pyometra

Thirty-nine (35%) of the dogs were diagnosed with closed cervix pyometra whereas 72 dogs (65%) were diagnosed with open cervix pyometra. In dogs with open cervix the median age was 9.0 years and the median weight 26.0 kg. In dogs with closed cervix the median age was 9.6 years and the median weight 25.0 kg. The differences between the two groups were not significant (*p* = 0.69 and 0.24, respectively).

### Mortality and complications

Of the 111 dogs, five (4.5%) died, whereof one was euthanized because of mammary tumours and another because of stump pyometra and concurrent severe illness diagnosed 20 days postoperatively, and the three other died one day after surgery due to miscellaneous causes (Table 1). All these pyometra cases had open cervix.

**Table 1:** Cause of death in the five bitches that died in present study

Bitch no.	Closed/open cervix pyometra	Cause of death
1	open	Euthanasia due to concomitant mammary tumours
2	open	Euthanasia 20 days after surgery due to stump pyometra and severe illness
3	open	Died one day after surgery due to severe septic peritonitis
4	open	Died one day after surgery due to concomitant myocarditis and disseminated intravascular coagulation
5	open	Died one day after surgery due to uncertain reasons and concomitant peritonitis

Complications were found in 16 dogs (14%, 16/111). The complications were detected in 4 of the 39 dogs (10%) with closed cervix pyometra and in 12 of the 72 dogs (17%) with open cervix pyometra. The proportion of dogs with complications did not differ significantly between the two groups.

Peritonitis with associated uterine rupture was observed in four dogs prior to surgery, and in five dogs after surgery, peritonitis without obvious uterine rupture was observed. Other complications included postoperative wound infection, conjunctivitis, chronic pyelonephritis, urinary tract infection and myocarditis/disseminated intravascular coagulation (Table 2). Additionally, one dog with open cervix pyometra required resuscitation during surgery.

**Table 2:** Complications detected in 16 surgically treated bitches with pyometra with open or closed cervix

Type of complication	Closed cervix (n)	Open cervix (n)
Uterine rupture and peritonitis diagnosed prior to surgery	1	3
Postoperative peritonitis without obvious uterine rupture	1	4
Postoperative wound infection	1	1
Conjunctivitis	1	1
Chronic pyelonephritis	0	1
Urinary tract infection	0	1
Myocarditis and disseminated intravascular coagulation	0	1

### Prolonged postoperative hospitalization

The proportion and number of dogs that required prolonged hospitalization did not differ between dogs with open (36%, *n* = 25/69) and closed cervix pyometra (24%, *n* = 9/37) (*p* = 0.2). Most dogs (61%, *n* = 71/105) with or without sepsis (*n* = 33 and *n* = 38, respectively) required normal postoperative hospitalization (1–2 days). Reported prolonged postoperative hospitalization, defined as ≥ 3 days, did not differ between dogs with sepsis (39%, *n* = 24/62) and dogs without sepsis (23%, *n* = 10/43) (*p* = 0.09).

### Systemic inflammatory response syndrome

Sepsis (systemic inflammatory response syndrome, SIRS, induced by infection) was more common in dogs with closed cervix pyometra than in dogs with open cervix pyometra (*p* = 0.007) (Table 3).

**Table 3:** Case history data, clinical and laboratory findings, SIRS status and presence of prolonged hospitalization in dogs with open cervix pyometra or closed cervix pyometra with data recorded in each parameter

Variable	Open cervix pyometra /number of cases		Closed cervix pyometra /number of cases		*p* value*
	*n*	(%)	*n*	(%)	
Case history Anorexia	39/72	54	24/39	62	0.7
General physical condition					
Normal or mild depression	50/71	70	17/39	43	0.006
Moderate or severe depression	21/71	30	22/39	56	
Polydipsia	39/72	54	24/39	62	0.5
Polyuria	37/72	51	22/39	56	0.7
Vomiting	13/67	19	9/36	25	0.6
Physical examination Hydration status					
No or slight dehydration	31/71	44	14/38	37	0.8
Moderate or severe dehydration	40/71	56	24/38	63	
Abdominal pain on palpation	35/66	53	24/39	50	0.8
Haematology					
Leukocytosis	42/68	62	33/37	89	0.003
Neutrophilia	32/68	47	26/35	74	0.008
Monocytosis	36/70	51	30/37	81	0.003
SIRS positive status	36/71	51	30/39	77	0.007
Prolonged hospitalization (>=3 days)	25/69	36	9/37	24	0.2

*SIRS* Systemic inflammatory response syndrome

* Chi-square test/Fisher’s exact test

### Case history and physical examination findings

Moderately to severely depressed general condition was more common in dogs with closed cervix compared to dogs with open cervix (*p* = 0.006) (Table 3). Anorexia, polydipsia/polyuria, vomiting, and moderate to severe dehydration did not differ significantly between the two groups (*p* ≥ 0.05) (Table 3).

### Hematology and serum biochemistry test results

Leukocytosis, neutrophilia and monocytosis were more common in dogs with closed cervix pyometra compared to dogs with open cervix pyometra (*p* = 0.003, *p* = 0.008, and 0.003, respectively) (Table 3). Furthermore, total white blood cell count (WBC), segmented neutrophils and monocyte numbers were significantly higher in dogs with closed cervix pyometra compared to dogs with open cervix pyometra (*p* < 0.05) (Table 4).

**Table 4:** Clinical and laboratory findings in 72 dogs with open cervix pyometra and 39 dogs with closed cervix pyometra

	Open cervix pyometra Median (interquartile range)	Closed cervix pyometra Median (interquartile range)	*p* value *	Reference range^a^
Hb (g/L)	122.0 (113.0-144.0)	137.0 (116.0-152.0)	0.1	132.0-199.0
PCV (%)	35.0 (32.0-40.0)	38.5 (32.0-43.0)	0.1	38.0-57.0
WBC (×10^9^/L)	16.9 (10.8-27.0)	23.1 (18.0–33.7)	0.006	5.8–16.0
Neutrophils (×10^9^/L)	10.9 (7.4–16.8)	15.5 (11.1–21.8)	0.005	3.0–11.5
Band neutrophils (×10^9^/L)	1.6 (0.6–5.4)	2.3 (1.2–4.4)	0.5	0.0–0.3
Lymphocytes (×10^9^/L)	1.4 (0.9–2.1)	1.4 (1.0–2.3)	0.2	1.4–4.8
Monocyte (×10^9^/L)	1.6 (0.7–3.4)	2.2 (1.7–4.0)	0.008	0.2–1.4
Eosinophils (×10^9^/L)	0.2 (0–0.5)	0.1 (0–0.4)	0.8	0.1–1.2
Basophils (×10^9^/L)	0	0	0.2	0.0–0.1
Bile acids (μmol/L)	1.8 (1.2–4.3)	3.7 (1.7–10.0)	0.2	0.0-12.0
ALT (μkat/L)	0.4 (0.3–0.6)	0.3 (0.2–0.4)	0.2	0.0–1.3
ALP (μkat/L)	3.4 (1.9–5.1)	4.3 (2.1–6.4)	0.3	<5.0
ALB (g/L)	26.0 (22.0-28.0)	26.0 (24.0-30.0)	0.2	29.0-39.0
Glucose (mmol/L)	5.1 (4.4–5.9)	4.7 (4.2–5.2)	0.06	4.5–5.8
BUN (mmol/L)	3.6 (2.8–5.5)	3.5 (2.6–5.2)	0.7	2.5–8.8
Serum creatinine (μmol/L)	67.0 (57.0-75.0)	74.0 (63.0-82.0)	0.1	40.0-130.0

*Hb* Haemoglobin, *PCV* Packed cell volume (haematocrit), *WBC* Total white blood cell count, *ALT* Alanine aminotransferase, *ALP* Alkaline phosphatase, *ALB* Albumin, *BUN* Blood urea nitrogen

*Wilcoxon Two Sample Test. ^a^Reference range at the Clinical Pathology Laboratory (University Animal Hospital, Swedish University of Agricultural Sciences, Uppsala)

## Discussion

Sixty-five percent of the dogs included the present study were diagnosed with open cervix pyometra. That open cervix is more common than closed cervix in pyometra is in accordance with the results of our previous study in which over three-quarter of dogs with pyometra were diagnosed as open cervix pyometra [9]. In the present study, sepsis was diagnosed in 59% of the dogs, i.e. the majority of pyometra cases, which is in agreement with other studies of dogs with the disease using the same criteria [3, 18, 19]. Furthermore, sepsis was more common in dogs with closed cervix pyometra compared to open cervix pyometra. These results support the general belief that dogs with pyometra without vaginal discharge are more severely ill compared to dogs with open cervix pyometra and vaginal discharge [17]. Sepsis was, however, not associated with worst outcome as measured by prolonged postoperative hospitalization or presence of complications in the present study. This finding is not in line with a study in dogs with pyometra in which SIRS was associated with prolonged hospitalization after surgical treatment [3]. Sepsis has also been associated with a poorer outcome i.e. increased hospitalization or higher risk of mortality in humans [20, 21]. It is difficult to explain the different results in studies of pyometra, because the same criteria were used to define dogs with sepsis. However, the selected criteria for SIRS with a sensitivity of 97% and specificity of 64% [22], may lead to the inclusion of 36% non-septic dogs in the septic group, and more specific SIRS criteria or a defined gold standard for diagnosing sepsis would be valuable for future studies.

In dogs with closed cervix pyometra, a moderately to severely depressed general condition was more commonly detected at admission than in dogs with open cervix. This might indicate that closed cervix pyometra would be more likely to have a poor outcome, because previously it was shown that dogs with pyometra and moderately to severely depressed general condition had a seven-fold increased risk of having a prolonged postoperative hospitalization [9]. More severely depressed general condition has also been associated with septic peritonitis [9]. Despite closed cervix not being associated with prolonged postoperative hospitalization or presence of complications in the current study, it is important to be aware that a more severe disease in closed cervix pyometra cases at admission can be expected.

Leukocytosis, neutrophilia and monocytosis were more commonly found in dogs with closed cervix pyometra, reflecting the higher amount of inflammatory response initiated by disease. Not only the amount but also the duration of leukocytosis may indicate prognosis because in human patients with persistent leukocytosis there is a high risk of developing infection [23, 24]. Furthermore, leukocytosis has been shown to indicate poor outcome as measured by mortality and duration of hospitalization whether with or without infection in both humans and dogs [25–28]. However, leukopaenia has also been shown to be associated with a peritonitis and prolonged hospitalization in dogs with pyometra [9].

The serum concentrations of creatinine and BUN did not differ significantly between the two groups and most dogs were within the normal range for both analyses. That in the present study only a few dogs with the disease were extremely ill and had elevated serum creatinine concentrations is in agreement with findings in other studies of dogs with the disease [9, 11, 15, 18]. None of the other clinical parameters analyzed differed significantly between the two groups in the present study.

The mortality in the surgically treated dogs was 4.5% (*n* = 5/111) which was slightly higher than in our previous study where 1% of 315 surgically treated pyometra dogs died [9]. This difference between studies might reflect individual differences in disease severity among the included dogs. Peritonitis was the most common complication in the dogs with pyometra studied here, present in 10% of the bitches, which is in agreement with the proportion reported in our previous study [9].

A limitation of the present study was the number of included dogs, because a larger study is necessary to identify minor differences in variables between the groups and for rarer variables such as mortality. Data from 22 dogs were also included in our previous retrospective study of 356 dogs, i.e. the results in both these studies are not completely independent. Grading of the general physical condition is somewhat subjective and was performed by several veterinarians filling in a similar protocol, which also is a study limitation.

## Conclusion

Dogs with closed cervix pyometra were more severely affected by the disease compared to dogs with open cervix pyometra as indicated by the more common finding of sepsis, leukocytosis, neutrophilia, monocytosis, and having moderately to severely depressed general condition in this group. Furthermore, total white blood cell count (WBC), segmented neutrophils and monocyte numbers were significantly higher in dogs with closed cervix pyometra. The present study thus provides, for the first time, data showing that closed cervix in dogs with pyometra is associated with more severe general illness at admission but not with increased postoperative hospitalization. This information might be valuable in clinical practice so that veterinarians are aware of a potentially more severe disease in dogs with closed cervix pyometra and that more intensive monitoring and treatment might be necessary to ensure a favourable outcome also in these dogs.

## Methods

### Study design

Clinical data, previously collected for two research studies were used for the analyses.

### Animals

Prospectively collected data from 111 female dogs diagnosed with pyometra at the University Animal Hospital (UDS), Swedish University of Agricultural Sciences (SLU), Uppsala, Sweden, intermittently during 2005–2012 was included.

The veterinarian in charge performed a complete physical examination and recorded case history and clinical examination data in a special study form which indicated the criteria categories for assessment. Case history, physical examination findings, laboratory test results together with evaluation by diagnostic imaging (radiography or/and ultrasonography) indicated a preliminary diagnosis of pyometra. All dogs were admitted to and treated by ovariohysterectomy at the University Animal Hospital (UDS), Swedish University of Agricultural Sciences (SLU), Uppsala. Written owner consent and daytime admission (Clinical Pathology Laboratory access) was a prerequisite for inclusion in the study. The definite diagnosis of dogs with pyometra was based on postoperative macroscopic identification of a pus-filled uterus, positive bacterial culture from the uterine content and histopathological examination of formaldehyde-fixated uteri and ovaries. Bitches with a histopathological diagnosis of mucometra, hydrometra or cystic endometrial hyperplasia were excluded [2].

Body temperature (BT), heart rate (HR), respiratory rate (RR), mucus membrane color, capillary refilling time (CRT), abdominal pain on palpation (absent, mild, moderate or severe), its location, hydration status and general physical condition/appearance (normal i.e. bright, alert and responsive; mildly depressed, moderately depressed, severely depressed or very severely depressed/non responsive as determined by the veterinarian in charge and also with consideration of the owner’s statement of how the dogs normally appears and acts) were recorded at the time of admission.

After surgical treatment, dogs with pyometra generally require postoperative care and monitoring at the UDS, SLU, for 1–2 days provided that no complications occur. Postoperative hospitalization of 3 days or more, because of specific complications or depressed general physical condition was defined as prolonged.

### Blood collection and laboratory analyses

#### Haematological and, biochemical analyses

Blood samples were aseptically collected for haematology and clinical biochemistry from the distal cephalic vein and transferred into EDTA and non-additive collection tubes (Vacutainer^®^, Becton-Dickinson, Stockholm, Sweden). The centrifugation was performed by using the blood collected in the non-additive tubes. The separated serum was used for analysing clinical biochemistry parameters. Clinical biochemical included Bile acids, Alanine aminotransferase (ALT), Glucose, Blood urea nitrogen (BUN), and Creatinine were measured (Abbott Architect c4000, Abbott Park, IL, USA). Haematological analyses (WBC including differential counts, haematocrit (PCV) and haemoglobin (Hb)) were performed (Advia 2120; Siemens Healthcare Diagnostics, Deer-field, IL, USA). Colorimetric method (bromocresol green) was used to analyse albumin and measured by using an automated analyser (Abbott Architect c4000, Abbott Park, IL, USA). All laboratory analyses were performed according to the routine methods at the Clinical Pathology Laboratory, UDS, SLU, Uppsala, Sweden.

### Definition of the systemic inflammatory response syndrome

Systemic inflammatory response syndrome, SIRS, was classified using the criteria defined by Hauptman and others [22]. Dogs fulfilling two or more of the following criteria were defined as SIRS positive for the purpose of data analyses (1): Body temperature (BT) < 38.1 °C (100.4 °F) or > 39.2 °C (102.6 °F); (2) Heart rate (HR) > 120 beats per min; (3) Respiratory rate (RR) > 20 breaths per minute; and Total white blood cell count (WBC) < 6 or > 16 × 10^3^/μL, or percentage band neutrophils (PBN) > 3%.

### Statistical analyses

The SAS program (version 9.3, SAS Institute Inc., Cary, NC, USA) was used for all analyses. Univariable associations between case history, physical examination and laboratory data, risk for prolonged hospitalization and risk of developing SIRS were analysed by Chi-Square test and Fisher’s exact test for comparing between dogs with closed or open cervix pyometra. For continuous variables, the Wilcoxon Two Sample Test was used to test the difference between the two groups. Dogs with moderately to severely depressed general physical condition were grouped together in the analyses due to few dogs with severe depression and none with very severe depression or that were non-responsive. *P* < 0.05 was set as significance level for differences between the two groups.
